# Hierarchical learning of gastric cancer molecular subtypes by integrating multi‐modal DNA‐level omics data and clinical stratification

**DOI:** 10.1002/qub2.45

**Published:** 2024-05-13

**Authors:** Binyu Yang, Siying Liu, Jiemin Xie, Xi Tang, Pan Guan, Yifan Zhu, Xuemei Liu, Yunhui Xiong, Zuli Yang, Weiyao Li, Yonghua Wang, Wen Chen, Qingjiao Li, Li C. Xia

**Affiliations:** ^1^ Department of Statistics and Financial Mathematics School of Mathematics South China University of Technology Guangzhou China; ^2^ School of Physics and Optoelectronics South China University of Technology Guangzhou China; ^3^ Department of Pathology The Sixth Affiliated Hospital Sun Yat‐sen University Guangzhou China; ^4^ School of Food Science and Engineering South China University of Technology Guangzhou China; ^5^ Department of Laboratory Medicine the Eighth Affiliated Hospital Sun Yat‐sen University Shenzhen China

**Keywords:** DNA alterations, gastric cancer, hierarchical classification, molecular subtyping, multi‐omics

## Abstract

Molecular subtyping of gastric cancer (GC) aims to comprehend its genetic landscape. However, the efficacy of current subtyping methods is hampered by their mixed use of molecular features, a lack of strategy optimization, and the limited availability of public GC datasets. There is a pressing need for a precise and easily adoptable subtyping approach for early DNA‐based screening and treatment. Based on TCGA subtypes, we developed a novel DNA‐based hierarchical classifier for gastric cancer molecular subtyping (HCG), which employs gene mutations, copy number aberrations, and methylation patterns as predictors. By incorporating the closely related esophageal adenocarcinomas dataset, we expanded the TCGA GC dataset for the training and testing of HCG (*n* = 453). The optimization of HCG was achieved through three hierarchical strategies using Lasso‐Logistic regression, evaluated by their overall the area under receiver operating characteristic curve (*auROC*), *accuracy*, *F*1 score, the area under *precision*‐*recall* curve (*auPRC*) and their capability for clinical stratification using multivariate survival analysis. Subtype‐specific DNA alteration biomarkers were discerned through difference tests based on HCG defined subtypes. Our HCG classifier demonstrated superior performance in terms of overall *auROC* (0.95), *accuracy* (0.88), *F*1 score (0.87) and *auPRC* (0.86), significantly improving the clinical stratification of patients (overall *p*‐value = 0.032). Difference tests identified 25 subtype‐specific DNA alterations, including a high mutation rate in the *SYNE*1, *ITGB*4, and *COL*22*A*1 genes for the MSI subtype, and hypermethylation of *ALS*2*CL*, *KIAA*0406, and *RPRD*1*B* genes for the EBV subtype. HCG is an accurate and robust classifier for DNA‐based GC molecular subtyping with highly predictive clinical stratification performance. The training and test datasets, along with the analysis programs of HCG, are accessible on the GitHub website (github.com/LabxSCUT).

**Abbreviations**
AUC: area under curve
CIN: chromosomal instability
CNA: copy number aberration
EA: esophageal adenocarcinomas
EBV: Epstein–Barr virus positive
GC: gastric cancer
GS: genomically stable
HCG: hierarchical classifier for gastric cancer molecular subtyping
II‐HC: two‐step hierarchical classification strategy
II‐HC(A): two‐step hierarchical classification strategy with all classes of alterations
II‐HC(C): two‐step hierarchical classification strategy with CNAs
II‐HC(G): two‐step hierarchical classification strategy with gene mutations
II‐HC(M): two‐step hierarchical classification strategy with methylations
III‐HC: three‐step hierarchical classification strategy
III‐HC(A): three‐step hierarchical classification strategy with all classes of alterations
III‐HC(C): three‐step hierarchical classification strategy with CNAs
III‐HC(G): three‐step hierarchical classification strategy with gene mutations
III‐HC(M): three‐step hierarchical classification strategy with methylations
I‐MC: one‐step multi‐class classification strategy
I‐MC(A): one‐step multi‐class classification strategy with all classes of alterations
I‐MC(C): one‐step multi‐class classification strategy with CNAs
I‐MC(G): one‐step multi‐class classification strategy with gene mutations
I‐MC(M): one‐step multi‐class classification strategy with methylations
LASSO: least absolute shrinkage and selection operator
MANOVA: multivariate analysis of variance
MSI: microsatellite instability
OS: overall survival
OVR: One‐vs‐Rest
ROC: receiver operating characteristic
SMOTE: synthetic minority oversampling technique
TCGA: The Cancer Genome Atlas

## 1 INTRODUCTION

Gastric cancer (GC) represents a significant global health challenge, with over one million new cases annually, ranking it fifth in incidence and third in mortality worldwide [1]. The precise subtyping of GC is essential for accurate diagnosis and prognosis, tumor staging, treatment guidance, recurrence monitoring, and drug development, as different subtypes exhibit distinct clinical outcomes and therapeutic responses [2, 3]. Despite its importance, GC subtyping faces significant challenges due to the disease’s complex etiology and high molecular heterogeneity [2, 4]. Therefore, the development of effective GC subtyping methods is both urgent and critical to enhance patient management and improve prognosis.

Prior to molecular subtyping, the classification of GC relied primarily on microscopic histological features. The Borrmann classification, which categorizes GC into four distinct subtypes (polypoid, fungating, ulcerated, and diffusely infiltrative carcinoma) based on gross appearance, is among the most prevalent methods [5]. Additionally, the Lauren classification distinguishes between two histological subtypes (intestinal or diffusive) based on microscopic characteristics [6]. However, these non‐molecular classifications have significant limitations in clinical practice, as the histological appearance may not fully reflect the biological diversity and molecular heterogeneity underlying the disease, rendering these classifications insufficient for guiding personalized therapy and precision medicine [7, 8, 9]. To reveal the underlying biological and molecular mechanisms of GC, researchers have shifted toward defining more precise, effective, and assessable GC subtypes at the molecular level [10, 11]. A landmark study by The Cancer Genome Atlas (TCGA) classified GC into four subtypes: chromosomal instability (CIN), genomically stable (GS), microsatellite instability (MSI), and Epstein–Barr virus positive (EBV) [11], based on an analysis of the genetic, epigenetic, and gene expression profiles of 295 primary GC samples. This study was pivotal, offering new perspectives for therapeutic development and led to numerous follow‐ups [12, 13, 14]. Despite these advances, a consensus on GC subtyping remains elusive.

The endeavor to develop an accurate, robust, and easily adoptable molecular subtyping classifier for GC faces multiple challenges. A primary issue is the undifferentiated and biologically ungrounded use of molecular features in current multi‐omics subtyping studies, which complicates data acquisition, integration, and analysis [15, 16, 17]. These studies often blend genomic and transcriptomic features without clear rationale. For instance, Röcken et al. integrated genomic and transcriptomic data to classify GC [18], while Liu et al. applied a residual graph convolutional network based on multi‐omics fusion data [19]. Notably, the TCGA subtyping study also encountered this problem. Such approaches overlooked the natural cascade of genetic information from the genome to transcriptome and proteome, with increasing noise perturbation at lower levels [20]. Thus, the mixed use of multi‐level features introduces unnecessary uncertainty into the resulting classifiers.

Secondly, the optimization of classification strategies in existing studies has not been rigorously pursued. Primarily, two types of subtyping strategies have been employed: one‐step multi‐class classification and multi‐step hierarchical classification. The one‐step multi‐class subtyping approach offers a straightforward method for identifying GC subtypes. For example, Zhou et al. classified GCs into three subtypes in a single step using 14 cancer functional states [21]. Li et al. clustered GCs into three subtypes (immunity‐deprived, stroma‐enriched, and immunity‐enriched) based on the activity of 15 pathways associated with immune, DNA repair, oncogenic, and stromal signatures [3]. However, these strategies do not account for the natural phylogeny of GC subtypes that result from cancer development and progression, potentially leading to an underutilization of this critical information for accurate subtyping. The hierarchical classification strategy has also been widely adopted. For example, the TCGA study sequentially categorized GC first by the presence of EBV features (EBV subtype), followed by the presence of MSI signatures (MSI subtype), and finally distinguished between genomically stable (GS subtype) and chromosomal instability (CIN subtype) subtypes based on the number of somatic copy number alterations (CNAs) [11]. Similarly, Tahara et al. classified GC into four subtypes, first by the CpG island methylator phenotype and subsequently by the presence of TP53 hotspot mutations [22]. However, these hierarchical strategies vary significantly in their structure, subgrouping, and group splitting. Moreover, the adoption of both multi‐class and hierarchical strategies has been largely subjective, lacking scientific benchmarking and justification. Therefore, there is a pressing need for a comprehensive and systematic method to evaluate these competing strategies, ensuring the development of more accurate and scientifically grounded subtyping methods.

Thirdly, the availability of public GC datasets for the development of a comprehensive multi‐feature subtype classifier has been limited. In single‐omics subtyping studies of GC, the most extensive methylation study was conducted by Zhang et al. using 398 GC samples [23], and the most extensive transcriptome study was by Cristescu et al., which analyzed gene expression data from 300 GC samples [24]. The largest multi‐omics public dataset is from the TCGA GC cohort, which included 383 samples with subtype information assessed by multi‐omics [25]. This highlights a significant need for more comprehensive datasets to improve our understanding of GC. To address this gap, we propose aggregating samples from related cancers, such as esophageal adenocarcinomas (EA), which typically occur in the lower portion of the esophagus [26] and exhibit molecular subtypes similar to those of GC, thereby increasing the pool of samples available for training and testing.

**FIGURE 1:**
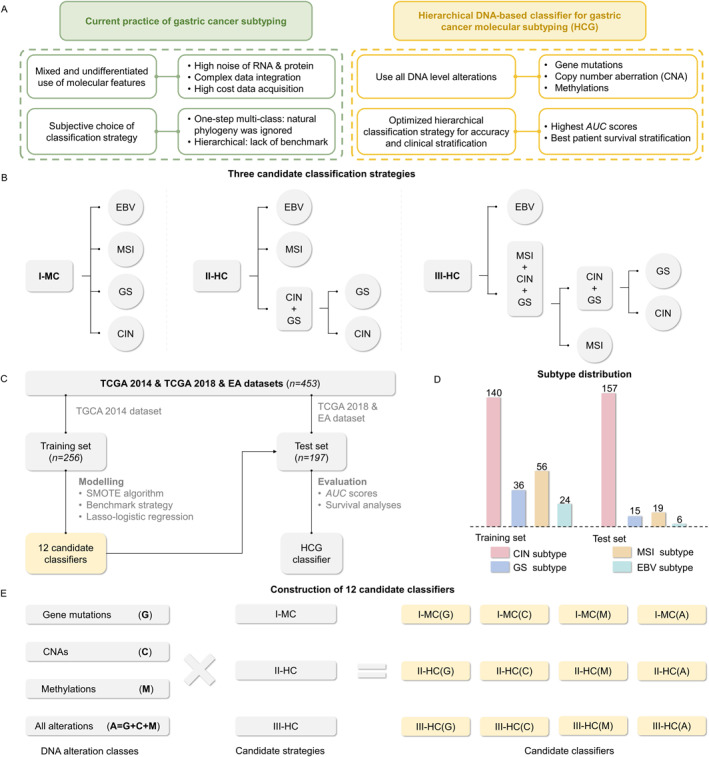
Study design, classification strategies and dataset description: (A) The conceptual design of DNA level gastric cancer subtyping; (B) Candidate classification strategies evaluated in this study; (C) Training and validating process; (D) Subtype distribution; (E) Enumeration of candidate classifiers.

In response to these challenges, we propose a novel approach to build a DNA alteration‐based GC subtype classifier, utilizing an expanded dataset and systematically evaluating potential classification strategies (Figure 1A). Our initiative to develop a comprehensive DNA‐level classifier is distinctive. Compared to mRNA and protein changes, DNA alterations are more fundamental, robust, and quantifiable. The central dogma dictates that genetic information flows from DNA to RNA and then to protein [27], suggesting that GC subtypes based on DNA features would be more fundamental for cancer cells. Moreover, DNA alterations are more reliably detected, given the structural stability of DNA molecules and their lower susceptibility to transcriptional and translational variations [28, 29, 30, 31]. However, previous studies on DNA‐based subtyping have not fully exploited the available DNA‐level information. For instance, Li et al. classified GC patients using only somatic mutational profiles and clinicopathological information [32], while Usui et al. relied solely on DNA methylation patterns to classify GCs into distinct molecular subtypes [33]. The partial utilization of DNA alterations in these studies likely overlooked critical features inherent in the types of alterations not considered. Therefore, exploring the potential for more accurate and robust classification of GC subtypes based on a comprehensive analysis of all available DNA alteration classes, including gene mutation, copy number aberration (CNA), and methylation alterations is both valuable and necessary.

Our research further endeavors to establish the criteria for evaluating the clinical efficacy and validity of competing molecular subtyping strategies. To ensure objectivity and eliminate potential biases in strategy selection, we conducted a comprehensive comparison of three candidate approaches (Figure 1B). This assessment was based on measures of classification performance, including overall *accuracy*, *F*1 score, and *AUC* score. Additionally, we evaluated the clinical relevance of the candidate classifiers, recognizing its importance for their effective application in clinical settings [34].

Moreover, our study seeks to expand the sample pool for omics‐based GC subtype modeling. By integrating the TCGA EA dataset with the TCGA GC dataset, we increased the number of samples available for model training and testing. Our approach is supported by previous research that has identified significant similarities between EA and GC subtypes. Specifically, both diseases originate from the epithelial cells lining the digestive tract and share common risk factors and symptoms [35]. Molecular features, such as CIN and TP53 mutations, have been observed in both EA and GC [36]. Furthermore, immunotherapy efficacy is similar for EA and GC, primarily for those expressing PD‐L1 or showing microsatellite instability [37]. To validate the integration of these datasets, we conducted a multivariate analysis of variance (MANOVA) test (see Materials and Methods), which showed no statistically significant differences between the CIN subtype and overall samples of the GC and EA datasets (*p*‐value = 0.9462 and 0.8934, respectively).

Our research culminated in the development of a novel DNA‐level GC molecular subtype classifier, termed HCG (hierarchical classifier for gastric cancer molecular subtyping) (Figure 1C). It was developed using a dataset of 453 combined GC and EA samples, incorporating all known DNA alterations (gene mutations, CNAs, and methylations) from the TCGA 2014, TCGA 2018, and EA datasets. The development process involved a rigorous evaluation of three classification strategies using Lasso‐Logistic regression and multivariate survival analysis, leading to the selection of an optimal two‐step hierarchical strategy. HCG demonstrated excellent overall performance (*auROC* = 0.95, *accuracy* = 0.88, *F*1 score = 0.87, and *auPRC* = 0.81) and the highest clinical stratification capacity (overall *p*‐value = 0.032). Based on the HCG subtypes, we performed difference tests and identified 25 statistically significant subtype‐specific DNA alterations.

## 2 RESULTS

### 2.1 DNA alterations alone sufficiently and accurately identify GC subtypes

The total samples (*n* = 453) analyzed in our study were first split into non‐overlapping training (TCGA 2014) and test (TCGA 2018 + EA) sets, as shown in Figure 1D. All the 12 DNA‐level classifiers (Figure 1E) were trained and cross‐validated using the training set augmented by the synthetic minority oversampling technique (SMOTE) algorithm [38], and then applied to the TCGA 2018 + EA test set. The *auROC* (area under receiver operating characteristic curve) scores, overall *accuracy*, *F*1 score, and *auPRC* (area under *precision*‐*recall* curve) scores were used as classification performance evaluation metrics. Please refer to the Materials and Methods section for details.

**FIGURE 2:**
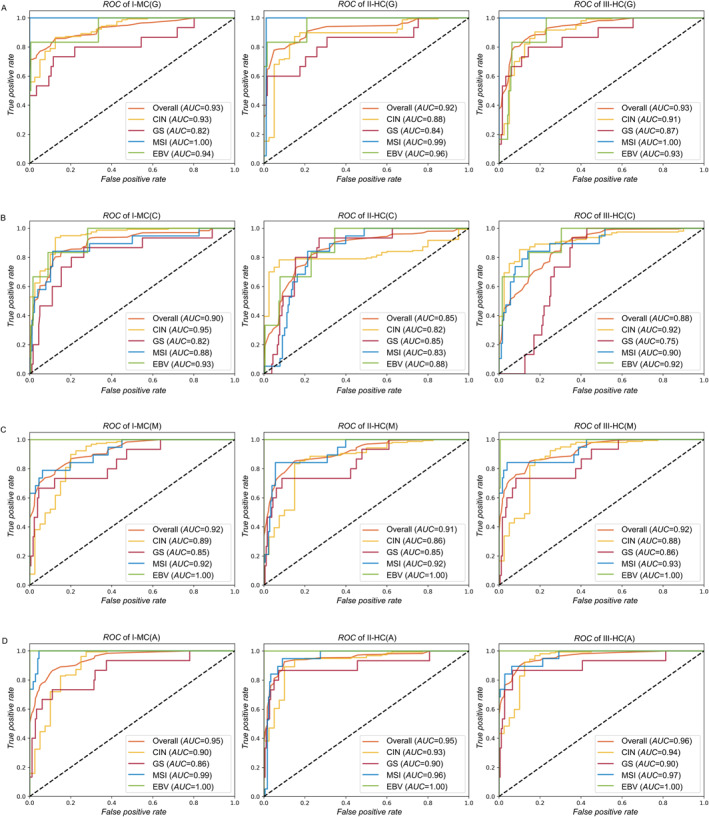
ROC characteristics of 12 candidate classifiers on test set: (A) I‐MC(G), II‐HC(G), III‐HC(G); (B) I‐MC (C), II‐HC(C), III‐HC(C); (C) I‐MC(M), II‐HC(M), III‐HC(M); (D) I‐MC(A), II‐HC(A), III‐HC(A).

We employed DNA‐level mutation, CNA, methylation, and their combination (all) as alteration classes to identify GC subtypes and evaluated their effectiveness using three candidate strategies (Figure 1E). The combination of input features and hierarchical learning strategies yielded 12 candidate classifiers, which were denoted as *S*_*i*_(*a*_*j*_), where *S*_*i*_ indicates the *i*th classification strategy (*i* ∈ {I‐MC, II‐HC, III‐HC}, which are abbreviations of one‐step multi‐class classification, two‐step hierarchical classification, and three‐step hierarchical classification, respectively) and *a*_*j*_ represents the *j*th input feature (*j* ∈ {G, C, M, A}, which are abbreviations of gene mutations (G), CNAs (C), methylations (M), and all alteration classes (A), respectively). For instance, I‐MC(G) denoted the classifier using gene mutations with the one‐step multi‐class (I‐MC) strategy and II‐HC(A) denoted the classifier using all classes of DNA alterations with the two‐step hierarchical classification (II‐HC) strategy. Our findings indicated that classifiers using all alteration classes outperformed those based on single classes in the test set. For example, with the I‐MC strategy, the overall *auROC* score for I‐MC(A) was 0.95, indicating a 0.02, 0.05, and 0.03 increase over I‐MC(G), I‐MC(C), and I‐MC(M) classifiers, respectively. Similarly, for the II‐HC strategy, the overall *auROC* score for II‐HC(A) was 0.95 (Figure 2D), indicating a 0.03, 0.10, and 0.04 increase over II‐HC(G), II‐HC(C), and II‐HC(M) classifiers, respectively. With the III‐HC strategy, the overall *auROC* score for III‐HC(A) was 0.96, indicating a 0.03, 0.08, and 0.04 increase over III‐HC(G), III‐HC(C), and III‐HC(M) classifiers, respectively. Consequently, the use of all alteration classes significantly enhanced the accuracy of GC subtype identification, overall *auROC* >0.95 in all subtypes. Similar results were obtained in terms of overall *accuracy*, *F*1 score, and *auPRC* metrics (see Table 1, Supplementary Table S1).

**TABLE 1:** Area under receiver operating characteristic curve, *accuracy*, *F*1 score and *auPRC* for I‐MC(A), II‐HC(A) and III‐HC(A) classifiers.

Classifiers	Subtypes	*auROC*		*Accuracy*		*F*1 score		*auPRC*	
		Score	Overall score	Score	Overall score	Score	Overall score	Score	Overall score
I‐MC(A)	CIN	0.9	0.95	0.91	0.86	0.93	0.82	0.96	0.85
	GS	0.86	0.95	0.73	0.86	0.61	0.82	0.52	0.85
	MSI	0.99	0.95	0.79	0.86	0.75	0.82	0.93	0.85
	EBV	1	0.95	1	0.86	1	0.82	1	0.85
II‐HC(A)	CIN	0.93	0.95	0.94	0.88	0.95	0.87	0.98	0.86
	GS	0.9	0.95	0.8	0.88	0.69	0.87	0.75	0.86
	MSI	0.96	0.95	0.79	0.88	0.83	0.87	0.7	0.86
	EBV	1	0.95	1	0.88	1	0.87	1	0.86
III‐HC(A)	CIN	0.94	0.96	0.94	0.87	0.95	0.85	0.98	0.88
	GS	0.9	0.96	0.8	0.87	0.67	0.85	0.65	0.88
	MSI	0.97	0.96	0.74	0.87	0.8	0.85	0.87	0.88
	EBV	1	0.96	1	0.87	1	0.85	1	0.88

In some instances, a single class of DNA alterations proved sufficient for accurate subtype prediction. For example, I‐MC(G), II‐HC(G), and III‐HC(G) strategies using gene mutations were notably effective in predicting the MSI subtype, achieving nearly perfect *AUC* scores of 1.00, 0.99, and 1.00, respectively (Figure 2A). However, the overall *auROC* scores of gene‐mutation only classifiers were significantly lower: 0.93, 0.88, and 0.91 for the CIN subtype; 0.82, 0.84, and 0.87 for the GS subtype; 0.94, 0.96, and 0.93 for the EBV subtype. These patterns were consistent across overall *accuracy*, *F*1 score, and *auPRC* metrics (see Table 1, Supplementary Table S1). This suggests that point mutations are the main alterations driving the development of the MSI subtype, which aligns with the nature of the MSI subtype‐arising from DNA mismatch repair (MMR) gene malfunctions and being characterized by extremely high level of point mutations [39].

Similarly, I‐MC(M), II‐HC(M), and III‐HC(M) strategies using methylations only achieved optimal performance when predicting the EBV subtype, all achieving a perfect *AUC* score of 1.00 (Figure 2C). In contrast, methylation‐based classifiers showed significantly lower overall *auROC* scores: 0.89, 0.86, and 0.88 for the CIN subtype; 0.85, 0.85, and 0.86 for the GS subtype; and 0.92, 0.92, and 0.93 for the MSI subtype. Similar results were obtained in terms of overall *accuracy*, *F*1 score, and *auPRC* metrics. The distinct methylation profiles of the EBV subtype, characterized by abnormally high levels of methylation, underscore the critical role of methylation features in defining the EBV subtype of GC, as corroborated by our previous studies [40].

### 2.2 Two‐step hierarchical classifier II‐HC(A) demonstrates the best clinical stratification

Considering the superior performance achieved by utilizing all classes of DNA alterations, we conducted a comparative analysis evaluating candidate classification strategies employing this comprehensive approach. Overall, the I‐MC(A), II‐HC(A), and III‐HC(A) classifiers all achieved highly competitive overall *auROC* scores (0.95, 0.95, and 0.96, respectively). This trend was consistent for subtype‐specific *AUC* scores as well (see Figure 2). Specifically, all classifiers obtained perfect *auROC* scores of 1.00 for the EBV subtype; 0.90, 0.93, and 0.94 for the CIN subtype; 0.86, 0.90, and 0.90 for the GS subtype; and 0.99, 0.96, and 0.97 for the MSI subtype, respectively (Figure 2D). Notably, the highest *auROC* scores were recorded for the EBV subtype, followed by MSI, and then CIN and GS subtypes, indicating that CIN and GS subtypes are the most challenging to accurately classify. Similar trends were observed in terms of overall *accuracy*, *F*1 score, and *auPRC* metrics (see Table 1 and Supplementary Table S1).

To assess the clinical relevance of the subtypes predicted by the original TCGA study and the I‐MC(A), II‐HC(A), III‐HC(A) classifiers, we conducted multivariate survival analyses that included the patient age and sex (see Materials and Methods). We set the reference as subtype = CIN, age<65, and sex = female. The results are presented in Table 2, with additional data on age and sex presented in Supplementary Table S2. Our analyses revealed that the II‐HC(A) classifier demonstrated superior clinical stratification capacity (overall *p*‐value = 0.032 for the multivariate Cox model, and a reduction of 0.019, 0.014, and 0.025 in comparison with TCGA, I‐MC(A), and III‐HC(A) defined subtypes, respectively). Specifically, the II‐HC(A) classifier achieved the most significant stratification for patients with the EBV subtype (*p*‐value = 0.221) and the second most significant stratification for patients with the GS and MSI subtypes (*p*‐value = 0.551 and 0.064, respectively). These results generally surpassed those of the TCGA study, which is considered the current gold standard. Based on the comprehensive evaluation of overall *auROC*, *accuracy*, *F*1 score, *auPRC*, and clinical relevance, the II‐HC(A) classifier was selected as the foundational classifier for HCG.

**TABLE 2:** Multivariate survival analyses using all samples from TCGA and I‐MC(A), II‐HC(A) and III‐HC(A) defined subtypes.

Classifier	Subtype	Count (%)	95% CI	Hazard ratio	*p*‐value^a^	Overall stratification *p*‐value^b^
TCGA	CIN (reference)	285 (66.6%)	/	/	/	0.051
	GS	47 (11.0%)	(0.60, 1.63)	0.99	0.973	0.051
	MSI	66 (15.4%)	(0.41, 1.01)	0.64	0.056	0.051
	EBV	30 (7.0%)	(0.41, 1.51)	0.79	0.468	0.051
I‐MC(A)	CIN (reference)	316 (73.8%)	/	/	/	0.046*
	GS	37 (8.6%)	(0.75, 2.04)	1.23	0.412	0.046*
	MSI	58 (13.6%)	(0.39, 1.04)	0.64	0.07	0.046*
	EBV	17 (4.0%)	(0.40, 2.07)	0.91	0.825	0.046*
II‐HC(A)	CIN (reference)	309 (72.2%)	/	/	/	0.032*
	GS	22 (5.1%)	(0.63, 2.39)	1.23	0.551	0.032*
	MSI	59 (13.8%)	(0.40, 1.01)	0.64	0.064	0.032*
	EBV	38 (8.9%)	(0.38, 1.22)	0.69	0.221	0.032*
III‐HC(A)	CIN (reference)	312 (72.9%)	/	/	/	0.057
	GS	23 (5.4%)	(0.64, 2.43)	1.24	0.524	0.057
	MSI	64 (15.0%)	(0.42, 1.10)	0.67	0.088	0.057
	EBV	29 (6.7%)	(0.42, 1.53)	0.80	0.495	0.057

^a^ Statistical significance is based on the fitted multivariate Cox model (log‐rank test).

^b^ Statistical significance is based on the fitted multivariate Cox model (likelihood ratio test).

*if *p* < 0.05.

### 2.3 HCG subtypes are linked to specific molecular features in GC

**FIGURE 3:**
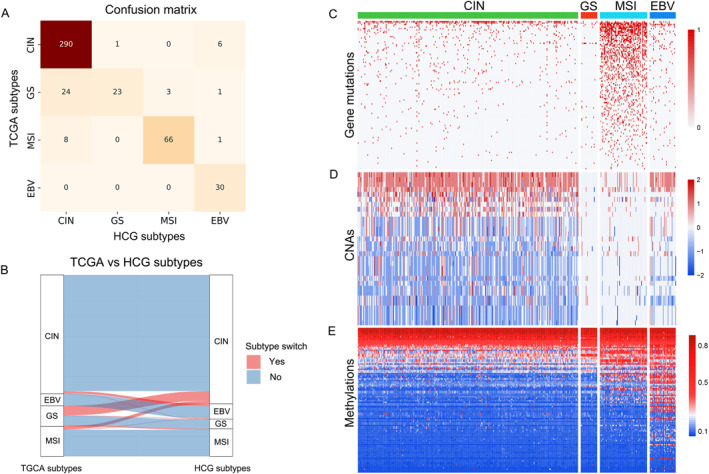
Analysis of HCG subtypes: (A) Confusion matrix between TCGA and HCG subtypes; (B) Alluvial plot between TCGA and HCG subtypes; (C–E) Heatmaps of all statistically significant gene mutations, copy number alterations, and methylations.

Our analysis revealed notable differences between HCG subtypes and TCGA defined subtypes. The confusion matrix and alluvial plot (Figure 3A,B) revealed that subtype‐switching occurred in 9.71% (44 out of 453) of the samples analyzed. Here, we denoted the HCG defined subtype X as HCG‐X and the TCGA defined subtype Y as TCGA‐Y. We observed that the TCGA‐EBV subtype remained consistent in the HCG classification. However, the TCGA‐GS subtype demonstrated the most significant transition, with 47.06% reclassified as HCG‐CIN, 5.88% to HCG‐MSI, and 1.96% to HCG‐EBV. Furthermore, a small fraction of the TCGA‐CIN subtype was reclassified as HCG‐GS (0.34%) and HCG‐MSI (2.02%). Similarly, 12% of the TCGA‐MSI subtype transitioned to HCG‐CIN (10.67%) and HCG‐EBV (1.33%).

The substantial shift of nearly 50% of the TCGA‐GS subtype to HCG‐CIN subtype may explain HCG’s significant improvement in clinical stratification. Previous studies have highlighted that CIN tumor cells suppress the immune response and exhibit greater chemotherapy sensitivity compared to GS tumor cells, which can lead to significant differences in treatment response, patient prognosis [3, 13]. Therefore, the more accurate identification of CIN and GS molecular subtypes using the HCG classifier could have significant clinical implications, potentially influencing treatment decisions and improving patient outcomes.

To visualize the genome‐wide DNA alteration patterns across subtypes, we generated a heatmap based on 247 DNA alteration features selected by HCG (Figure 3C–E). This analysis revealed that the HCG‐MSI subtype exhibited the highest frequency of mutations (Figure 3C), with mutation rates (see Materials and Methods) for the HCG‐CIN, GS, MSI, and EBV subtypes being 0.0123, $\underset{‾}{0.0044}$, $\bar{0.0998}$, and 0.0140, respectively. The elevated mutation rate in the MSI subtype supports the existing literature, which links the MSI subtype to a compromised DNA mismatch repair pathway, resulting in a high occurrence of point mutations [40]. Additionally, the HCG‐CIN subtype was found to have the highest CNA rate (see Materials and Methods), while the HCG‐GS subtype had the lowest (Figure 3D). The computed CNA rates for the HCG‐CIN, GS, MSI, and EBV subtypes were $\bar{0.544}$, $\underset{‾}{0.0245}$, 0.097, and 0.0433, respectively. This finding is consistent with previous studies that have associated the CIN subtype with extensive chromosomal loss or rearrangement. In contrast, the GS subtype is known for genomic stability, characterized by the absence of large‐scale CNAs [41, 42, 43]. Furthermore, we found that the HCG‐EBV subtype displayed hypermethylation characteristics (Figure 3E). The methylation rates (see Materials and Methods) for the HCG‐CIN, GS, MSI, and EBV subtypes were $\underset{‾}{0.254}$, 0.271, 0.281, and $\bar{0.326}$, respectively. Notably, only the HCG‐EBV subtype exhibited an elevated hypermethylation pattern, which greatly surpassed the average methylation rate of 0.266. This result is consistent with previous research that has identified extreme DNA hypermethylation as a hallmark of the EBV subtype [33, 40, 44].

### 2.4 The HCG classifier identifies novel subtype‐specific DNA alterations

Discovering subtype‐specific markers is crucial for developing targeted therapies. Taking advantage of the improved classification outcomes of HCG, we performed difference tests (see Materials and Methods) and identified 25 statistically significant subtype‐specific DNA alterations, as shown in Table 3. For the HCG‐CIN subtype, three CNA‐based (*CDH1*, *CDH3*, *KIF2*6B) and two methylation‐based (*CDR2*, *CUL4B*) DNA alteration markers were identified. These DNA alterations have previously been linked to GC in general. For example, the *CDH1* gene, which encodes E‐cadherin, is known for mutations that are among the most common germline mutations detected in GC, particularly associated with hereditary diffuse GC [45]. The *CDH3* gene, encoding P‐cadherin, has been found to be upregulated in GC [46]. *KIF2*6B encodes a kinesin family member and is significantly upregulated in metastatic GC samples [47]. *CUL4B* has been shown to promote cell invasion and epithelial–mesenchymal transition in vitro, as well as tumor growth and metastasis in vivo [48]. Our study is among the first to validate their CIN‐specific nature. Notably, the HCG classifier also identified *CDR2* hypomethylation as a CIN‐specific marker. While *CDR2* has previously been recognized as a tumor antigen in a substantial proportion of breast cancer [49], the mechanism behind *CDR2* and CIN subtype association remains to be explored.

**TABLE 3:** Novel subtype‐specific DNA alterations as identified by HCG.

	CIN	GS	MSI	EBV
Gene mutation	/	*NFASC*, *MYOM2*, *PTPRJ*, *PLXNB2* ^a^, *TTK*, *ECEL1* ^a^, *ARHGEF15* ^a^, *TRPM4* ^a^, *CYP4F2* ^a^, *CA10*	*SYNE1*, *ITGB4* ^a^, *COL22A1* ^a^	/
Copy number aberration	*CDH1* (+), *CDH3* (+), *KIF2*6B(−)	/	/	/
Methylation	*CDR2* (−)^a^, *CUL4B* (+)	*PRKCQ* (−)^a^, *IGF1* (−), *CRYGB* (−)^a^, *TSTD1* (+)^a^	/	*ALS2CL*(+)^a^, *KIAA0406* (+)^a^, *RPRD1B* (+)

*Note*: markers in each grid were ordered by statistical significance as found by difference tests. (+) denotes copy number amplification or hypermethylation. (−) denotes copy number deletion or hypomethylation.

^a^ Novel GC biomarkers by this study to our knowledge.

For the HCG‐GS subtype, ten mutation‐based (*NFASC*, *MYOM2*, *PTPRJ*, *PLXNB2*, *TTK*, *ECEL1*, *ARHGEF15*, *TRPM4*, *CYP4F2*, *CA10*) and four methylation‐based (*PRKCQ*, *IGF1*, *CRYGB*, *TSTD1*) subtype markers were identified. Notably, *TSTD1* hypermethylation was identified by the HCG classifier as a GS‐specific marker for the first time. Previous research has shown that *TSTD1* expression is significantly inversely correlated with *FZD1* expression, which in turn is positively correlated with *FZD2* and *FZD6*, all of which are linked to GC. Notably, elevated expressions of *FZDs* have been reported to inhibit the proliferation, migration, and invasion of GC cells through the activation of the non‐classical Wnt signaling pathway [50]. Therefore, the hypermethylation of *TSTD1* may reduce its expression, promoting the expression of *FZD* genes, which deserves further investigation to validate its role and mechanism.

For the HCG‐MSI subtype, our analysis identified three signature alterations, including high‐level of point mutations in the *SYNE1*, *ITGB4*, and *COL22A1* genes. These are consistent with the increased mutational load inherent to the MSI subtype [51]. Notably, the mutated *ITGB4* gene was newly identified by the HCG classifier as an MSI‐specific marker. In fact, the MSI subtype is driven by underlying defects in the DNA mismatch repair system [52], which results in various dysfunctions in pathways like Wnt/β‐catenin signaling, which influences cell proliferation [53]. It has been shown that *ITGB4* mutations decrease or impair its encoded integrin β4 subunit [54], which participates in the Wnt/β‐catenin signaling pathway by inhibiting Wnt‐responsive gene transcription and cell division [55]. Therefore, *ITGB4* mutations could diminish its expression or functionality, thus reducing its inhibitory effect on the Wnt signaling pathway [52, 56], and enhancing the MSI phenotype.

For the HCG‐EBV subtype, our analysis identified three signature alterations, including *RPRD1B*, *KIAA0406*, and *ALS2CL* hypermethylation, reflecting the high CpG island methylator phenotype of the EBV subtype [57]. The role of *RPRD1B*’s product in accelerating the G2/M transition, through interaction with Aurora kinase B and the ROS‐related p53 pathway that facilitates faster division of GC cells [58], suggests that its hypermethylation could act as a protective factor by suppressing cancer cell division, aligning with the better prognosis associated with the EBV subtype [13]. Similarly, the *KIAA0406* gene encodes a vital constituent of the TTT complex that is known to regulate the DNA damage response [59]. This implies that the hypermethylation of *KIAA0406* may impair the functionality of the TTT complex, allowing increased DNA damage and mutational burdens, consistent with the high mutational burdens observed in the EBV subtype (second highest mutation rate, following MSI) [57].

## 3 DISCUSSION

Molecular subtypes and signatures, associated with distinct clinical outcomes, become increasingly well‐characterized in solid tumors, paving the way for improved clinical management through precision‐driven treatment plans [60, 61, 62]. For GC, several molecular subtyping studies have been reported [10, 13, 32, 44, 63], yet a consensus on the most effective classification system remains elusive, underscoring the need for novel methodology development. In response, we developed an accurate, robust, and easily adoptable molecular subtype classifier HCG, which demonstrated exceptional performance across several metrics, including overall *auROC*, *accuracy*, *F*1 score, *auPRC* and significantly improved the clinical stratification of GC patients.

Our approach offers a more robust and reproducible approach to subtype identification in GC. While previous studies, such as the TCGA study [11] and many others [18, 19], have shown that integrating multi‐omics (including transient transcriptomics) data can yield relatively accurate predictions, our findings underscore the sufficiency of using stable DNA‐level information for GC subtype classification. The *AUC* scores for the HCG classifier using all DNA alterations categories were all greater than 0.95. This superior performance is underpinned by biological principles, as DNA‐level genetics and epigenetics changes determine downstream phenotypes. For example, DNA methylation not only modulates chromatin structure but also plays a protein‐independent regulatory role by affecting DNA stiffness, influencing the genome’s 3D structure, and thereby regulating gene expression [64, 65]. Therefore, genome and epigenome aberrations are believed to synergistically interact and contribute to gastric carcinogenesis. However, further research and comprehensive analyses that integrate both genomic and epigenomic data are essential to fully understand the extent of this deterministic relationship.

Our study also underscores the importance of considering clinical relevance when evaluating subtyping classifiers, as the classification structure profoundly influences clinical relevance. For example, Pretzsch et al. established a classification system for GC molecular subtyping using immunohistochemistry and morphology‐based analyses as markers, which not only had clinical relevance but also reproduced the ACRG molecular subtypes of GC [66]. Similarly, Ramos et al. found that IHC/ISH analysis could distinguish immunophenotypic groups of GC with distinct characteristics and prognoses [67]. Our findings reveal variations in clinical relevance among different competitively performing classifiers. Specifically, survival outcomes were better stratified based on the HCG (II‐HC(A)) subtypes (overall *p‐value* = 0.032) compared to TCGA subtypes (overall *p*‐value = 0.051), while the stratification based on III‐HC(A) subtypes (overall *p*‐value = 0.057) were inferior to those of TCGA, though the II‐HC(A) and III‐HC(A) classifiers had indistinguishable *AUC* scores. This highlights the importance of accurate clinical stratification in influencing therapeutic decisions and patient outcomes [68], making it vital to assess classifiers with regards to clinical relevance.

Our study gave further evidence that molecular subtypes should be considered in the development of new GC drugs and therapies, given the significant subtype‐specific DNA‐level alterations observed. Clinical trials have shown the potential of MSI‐H status as a biomarker for pembrolizumab therapy, with MSI subtype patients not showing improvements in the median overall survival (OS) for both pembrolizumab monotherapy (95% CI, 10.7 months to not reached) and pembrolizumab plus chemotherapy (95% CI, 3.6 months to not reached) compared to a median OS of 8.5 months (95% CI, 5.3–20.8 months) for chemotherapy alone. The early divergence of the survival curves between patients with MSI who received pembrolizumab therapy versus chemotherapy suggests that earlier introduction of pembrolizumab is beneficial for MSI subtype patients [69]. Additionally, several studies suggested that chemotherapy might not be the optimal treatment choice for patients with the MSI subtype. Previous research has demonstrated that patients with the MSI subtype respond worse to chemotherapy alone [70] and some who underwent neoadjuvant chemotherapy experience adverse reactions. Even those who responded to neoadjuvant chemotherapy did not experience enhanced prognoses compared to non‐responders [71], underscore the need for improved patient subtyping to guide and enhance GC treatment.

Nonetheless, our study faced limitations, including the limited public data available for analysis. Despite our attempts to address the inadequacy of available data by incorporating the TCGA EA samples, the need for more comprehensive data remains. Furthermore, sample imbalance, particularly with most EA samples being of the CIN subtype, was mitigated by SMOTE balancing in training, but further studies targeting minor subtypes are required. Additionally, integrating features such as chromatin accessibility could provide deeper insights into the functional consequences and regulatory mechanisms underlying GC subtyping. Although the limited availability of context‐specific datasets hindered the direct incorporation of chromatin accessibility data in our study, we acknowledge its importance in gene regulation [72, 73]. Future research can explore adjusting gene weights or introducing modifications based on chromatin accessibility data to enhance our understanding of GC subtypes. Lastly, our HCG classifier, based on the four TCGA‐defined GC molecular subtypes, may not accommodate other subtype classification schemes, highlighting the potential for future models to consider additional GC molecular subtype classification methods [3, 10] as more data become available.

## 4 MATERIALS AND METHODS

### 4.1 Data source and pre‐processing

The genomic data analyzed in this study, including gene mutations, CNAs, methylation alterations, and clinical data, were obtained from the cBioPortal database [74]. The sample labels of CIN, GS, MSI, and EBV molecular subtypes were derived from the TCGA GC study in 2014 and denoted as TCGA 2014. Furthermore, an additional dataset from the TCGA PanCanAtlas published in 2018 was included and denoted as TCGA 2018. The TCGA dataset that included EA samples were denoted as EA (Figure 1C).

TCGA 2014 was used as the training set, while TCGA 2018 and EA were used as test sets. To pre‐process the mutation data, the downloaded mutation profiles were mapped to the gene level using a Boolean matrix, where each cell indicated whether the *i*th gene was mutated at least once in the *j*th patient. For CNAs, copy number values downloaded from cBioPortal were mapped to the gene level. For methylation data, missing values were filled with the nearest 10 neighbors by applying the *knnImputation()* function in R’s *DMwR* package in cases where no more than half were missing [75, 76, 77]. The implemented approach employed the K‐nearest neighbors (KNN) imputation method. Specifically, for each sample with missing values, the KNN imputation method identified the KNN within the dataset. Subsequently, the missing values were imputed by replacing them with the average values derived from the “*k*” neighbors identified. Notably, the KNN imputation technique relied on the Euclidean distance matrix to effectively determine the closest neighbors, thereby facilitating the estimation of the missing values present within the dataset. In this study, we chose *k* = 10 for the KNN imputation method.

Genes corresponding to multiple methylation values were then assigned the averaged value. Finally, we standardized each alteration using min–max Scaling, as shown in the following formula:

(1) $$x_{\text{scaled}}=\frac{x-\min\left(x\right)}{\max\left(x\right)-\min\left(x\right)}$$

where *x* represents the value of each alteration, while min(*x*) and max(*x*) denote the minimum and maximum values of the alteration across all samples, respectively.

### 4.2 SMOTE algorithm

To address the issue of imbalanced distribution in the four subtypes (shown in Figure 1D) and to mitigate potential biases, we applied a data augmentation technique called SMOTE prior to training [38]. Specifically, we increased the number of samples for the GS, MSI, and EBV subtypes in the training set by generating synthetic samples using the K‐nearest neighbor method. This approach resulted in an increased number of training samples (balanced for 140 samples for each subtype) and reduced the risk of overfitting in the constructed model. The SMOTE algorithm was implemented using the *imblearn* library in Python.

### 4.3 Lasso‐Logistic regression

We applied the Lasso‐Logistic regression model to solve the classification problem, consisting of least absolute shrinkage, selection operator (Lasso), and the logistic regression methods, which were implemented in Python through the scikit‐learn library. Lasso is an efficient approach to model high‐dimensional and multi‐modal data, while utilizing soft thresholding to avoid overfitting, which selects significant alteration features by regularizing the regression coefficients of non‐informative features to zeros. While implementing the model, we performed a 5‐fold cross‐validation. For the regularization weight *λ* (lambda), we chose the minimal lambda that achieves the highest classification prediction *AUC* score.

In details, suppose the response variable has *K* levels *G* = {1,2,…,*K*}, and each sample *x*_*i*_ in our study has *m* (*m* = 53,496) features, that is, *x*_*i*_ = (*x*_*i*,1_,*x*_*i*,2_,…,*x*_*i*,*m*_), then we model:

(2) $$\mathrm{Pr}\;\left(G=k|X=x\right)=\frac{e^{\beta_{0k}+\beta_{k}^{T}x}}{\sum_{l=1}^{K}e^{\beta_{0l}+\beta_{l}^{T}x}}$$

Let *Y* be the *N* × *K* indicator response matrix, with elements *y*_*il*_ = *I* (*g*_*i*_ = *l*). Then, the Lasso elastic net penalized negative log‐likelihood function of logistic regression becomes:

(3) $$l\;\left({\left\{\beta_{0k},\beta_{k}\right\}}_{1}^{K}\right)=-[\frac{1}{N}\sum_{i=1}^{N}(\sum_{k=1}^{K}y_{il}\left(\beta_{0k}+x_{i}^{T}\beta_{k}\right)-\log\left(\sum_{l=1}^{K}e^{\beta_{0l}+x_{i}^{T}\beta_{l}}\right))]+\lambda \sum_{j=1}^{p}\left|\beta_{j}\right|$$

where *β* is a *p* × *K* matrix of coefficients, *β*_*k*_ refers to the *k*th column (for outcome category *k*), and *β*_*j*_ the *j*th row (vector of *K* coefficients for variable *j*), *λ* is the penalty parameter as chosen by previous cross‐validation.

### 4.4 Evaluation metrics

The performances of the 12 candidate classifiers were assessed by overall *accuracy*, *F*1 score, the *auPRC*, and the *auROC*.

The overall *accuracy* is a commonly used performance metric in classification tasks, which measures the proportion of correctly classified instances out of the total number of instances. The *accuracy* can be calculated as follows:

(4) $$\mathrm{Accuracy}=\frac{\text{Number}\;\text{of}\;\text{correct}\;\text{predictions}}{\text{Total}\;\text{number}\;\text{of}\;\text{predictions}}$$

The *F*1 score combines *p*recision and *r*ecall into a single value and measures the balance between *precision* and *recall*. *Recall* quantifies the model’s capability to correctly identify positive instances out of all actual positive instances. *Precision* is a measure of the model’s ability to accurately identify positive instances from the total predicted positive instances. The formula for *recall* is given by:

(5) $$Recall=\frac{\mathrm{True}\;\mathrm{positives}}{\mathrm{True}\;\mathrm{positives}+\mathrm{False}\;\mathrm{negatives}}$$

(6) $$Precision=\frac{\mathrm{True}\;\mathrm{positives}}{\mathrm{True}\;\mathrm{positives}+\mathrm{False}\;\mathrm{positives}}$$

(7) $$F1=\frac{2\times Precision\times Recall}{Precision+Recall}$$

The *precision‐recall* curve is a graphical representation that illustrates the trade‐off between *precision* and *recall* for different classification thresholds. It plots *precision* on the *y*‐axis and *recall* on the *x*‐axis. The curve shows how the *precision* and *recall* values change as the threshold for classifying instances varies. The formulas are given by:

(8) $$auPRC=\int_{0}^{1}Precision\left(r\right)\;dRecall\left(r\right)$$

where *precision*(*r*) is the *precision* at a specific *recall* level *r*.

The *ROC* curve is a graphical representation that illustrates the *true positive rate* (*TPR*) on the *y*‐axis against the *false positive rate* (*FPR*) on the *x*‐axis at different thresholds. The *AUC* score can be used to evaluate the general performance of a classifier. The formulas are given by:

(9) $$TPR=\frac{\mathrm{True}\;\mathrm{positives}}{\mathrm{True}\;\mathrm{positives}+\mathrm{False}\;\mathrm{negatives}}$$

(10) $$FPR=\frac{\mathrm{False}\;\mathrm{positives}}{\mathrm{False}\;\mathrm{positives}+\mathrm{True}\;\mathrm{negatives}}$$

(11) $$auROC=\int_{0}^{1}TPR\left(f\right)\;dFPR\left(f\right)$$

where *TPR*(*f*) and *FPR*(*f*) are given at different threshold *f*.

While the *auPRC* and *auROC* are primarily used to evaluate binary classification, they can be extended to multi‐class classification using the One‐vs‐Rest (OVR) strategy. This approach involves treating each class as the positive class while all other classes are considered the negative. By employing this OVR method, we generated a *PRC* and *ROC* curve for each class, from which the classifier’s ability to distinguish a particular class can then be assessed by its corresponding *AUC* score. The overall performance of the classifier was evaluated by calculating the average of the *AUC* scores for each class.

### 4.5 Survival analysis

To evaluate the clinical stratification ability of the classifiers, survival analyses were performed in R using the *survival* and *survminer* package with OS as the primary endpoint. In univariate analysis, the log‐rank test was used to compare survival outcomes and Kaplan—Meier (KM) curves were generated to provide a visual representation of the survival data.

In multivariate analysis, patient age and sex were included as a covariate. Cox proportional hazards regression was used to analyze the effect of subtypes, age, and sex, using the *coxph()* R function. The forest plot (organized into Table 2 and Supplementary Table S2) showed survival outcomes with subtype = CIN, age<65, and sex = male as reference. Analyses involving multiple comparisons were adjusted using the Benjamini‐Hochberg (BH) False Discovery Rate (FDR) method, which controls the FDR with the Holm–Bonferroni method for multiple hypothesis testing. All statistical tests were determined to be significant based on the BH‐corrected significance level of 0.05.

### 4.6 Mutation, CNA, and methylation rates

For mutation rate, we first computed the average mutation rate of each sample across all genes. Then, we used the HCG classifier to categorize the samples into *k* subtype (*k* ∈ {*CIN*,*GS*,*MSI*,*EBV*}) and calculated the average mutation rate of each subtype. Let *mu*_*i*,*j*_ be the mutation count for sample *i* in gene *j* and *n*_*mu*_ be the total number of genes in mutation profiles. The mutation rate for the *k*th subtype is:

(12) $$\text{Mutation}\;\text{rat}e_{k}=\frac{\sum_{i\in S_{k}}\left(\sum_{j=1}^{n_{mu}}mu_{i,j}\right)}{n_{k}\cdot n_{mu}}$$

where *S*_*k*_ is the set of the *k*th subtype and *n*_*k*_ is the number of samples that belong to the *k*th subtype.

Similarly, we defined the CNA and methylation rates in Equations (6) and (7). Let *c*_*i*,*j*_ and *me*_*i*,*j*_ be the CNA count and methylation level for sample *i* in gene *j*, respectively, *n*_*CNA*_ and *n*_*me*_ be the total number of genes in CNA and methylation profiles, respectively. Then, the CNA rate and methylation rate for the *k*th subtype are:

(13) $$\text{CNA}\;\text{rat}e_{k}=\frac{\sum_{i\in S_{k}}\left(\sum_{j=1}^{n_{CNA}}1_{\left\{c_{i,j}\neq 0\right\}}\right)}{n_{k}\cdot n_{CNA}}$$

(14) $$\text{Methylation}\;\text{rat}e_{k}=\frac{\sum_{i\in S_{k}}\left(\sum_{j=1}^{n_{me}}me_{i,j}\right)}{n_{k}\cdot n_{me}}$$

where 1 (·) is the indicator function.

### 4.7 Difference test

To identify significant subtype‐specific DNA alterations, difference tests were conducted in R on 247 DNA alterations selected by the HCG classifier. For mutation data, the Fisher’s exact test was applied using the *fisher*.*test()* R function. For copy number aberrations and methylation data, the independent Student’s *T*‐test was employed using the *t*.*test* () R function. DNA alterations were considered significantly different if the adjusted *p*‐value (FDR correction method) was less than 0.00001 using a two‐sided test with the OVR strategy. Finally, we performed duplicate gene removal.

To compare differences between the CIN subtype of GC and EA datasets, we conducted MANOVA using the *manova()* R function. The MANOVA test allows for the simultaneous analysis of multiple dependent variables and is used to determine if there are any significant differences between groups based on multiple response variables.

## AUTHOR CONTRIBUTIONS

Binyu Yang, Siying Liu, Jiemin Xie, Xi Tang, Qingjiao Li and Li C. Xia conceived and designed the study. Siying Liu and Yifan Zhu collected the data. Binyu Yang, Siying Liu, and Pan Guan performed the modeling and analysis. Binyu Yang, Siying Liu, Jiemin Xie, Qingjiao Li and Li C. Xia wrote the manuscript. All authors edited the manuscript and approved the submitted version.

## CONFLICT OF INTEREST STATEMENT

The authors Binyu Yang, Siying Liu, Jiemin Xie, Xi Tang, Pan Guan, Yifan Zhu, Xuemei Liu, Yunhui Xiong, Zuli Yang, Weiyao Li, Yonghua Wang, Wen Chen, Qingjiao Li and Li C. Xia declare that they have no conflict of interest or financial conflicts to disclose.

## ETHICS STATEMENT

The article does not contain any human or animal subjects performed by any of the authors.

## Supporting information

Supporting Information S1
