# Supplementary material for: Hierarchical learning of gastric cancer molecular subtypes by integrating multi‐modal DNA‐level omics data and clinical stratification
Source: Quant Biol. 2024 May 13;12(2):182–96. doi: 10.1002/qub2.45 (PMC12806395; doi:10.1002/qub2.45)
Supplement: Supplementary file 1 — Supporting Information S1 [file QUB2-12-182-s001.docx]

**Supplementary Materials**

**Hierarchical learning of gastric cancer molecular subtypes by integrating multi-modal DNA-level omics data and clinical stratification**

Binyu Yang^1,^**^†^**, Siying Liu^1,^**^†^**, Jiemin Xie^1,^**^†^**, Xi Tang^1,^**^‡^**, Pan Guan^1,^**^‡^**, Yifan Zhu^1,^**^‡^**, Xuemei Liu^2^, Yunhui Xiong^1^, Zuli Yang^3^, Weiyao Li^3^, Yonghua Wang^4^, Wen Chen^4^, Qingjiao Li^5,*^, Li C. Xia^1,*^

^1^ Department of Statistics and Financial Mathematics, School of Mathematics, South China University of Technology, Guangzhou 510000, China

^2^ School of Physics and Optoelectronics, South China University of Technology, Guangzhou 510000, Guangdong, China

^3^ Department of Pathology, the Sixth Affiliated Hospital, Sun Yat-sen University, Guangzhou 510000, Guangdong, China

^4^ School of Food Science and Engineering, South China University of Technology, Guangzhou 510000, Guangdong, China

^5^ Department of Laboratory Medicine, the Eighth Affiliated Hospital, Sun Yat-Sen University, Shenzhen 518000, Guangdong, China

* Correspondence author. Qingjiao Li. E-mail: [liqj23@mail.sysu.edu.cn](mailto:liqj23@mail.sysu.edu.cn); Li C. Xia. E-mail: [lcxia@scut.edu.cn](mailto:lcxia@scut.edu.cn)

^†^ These authors are co-first authors.

^‡^ These authors contributed equally to this work.

**Supplementary Materials includes:**

1. Supplementary Figures

2. Supplementary Tables

**1. Supplementary Figures**

**
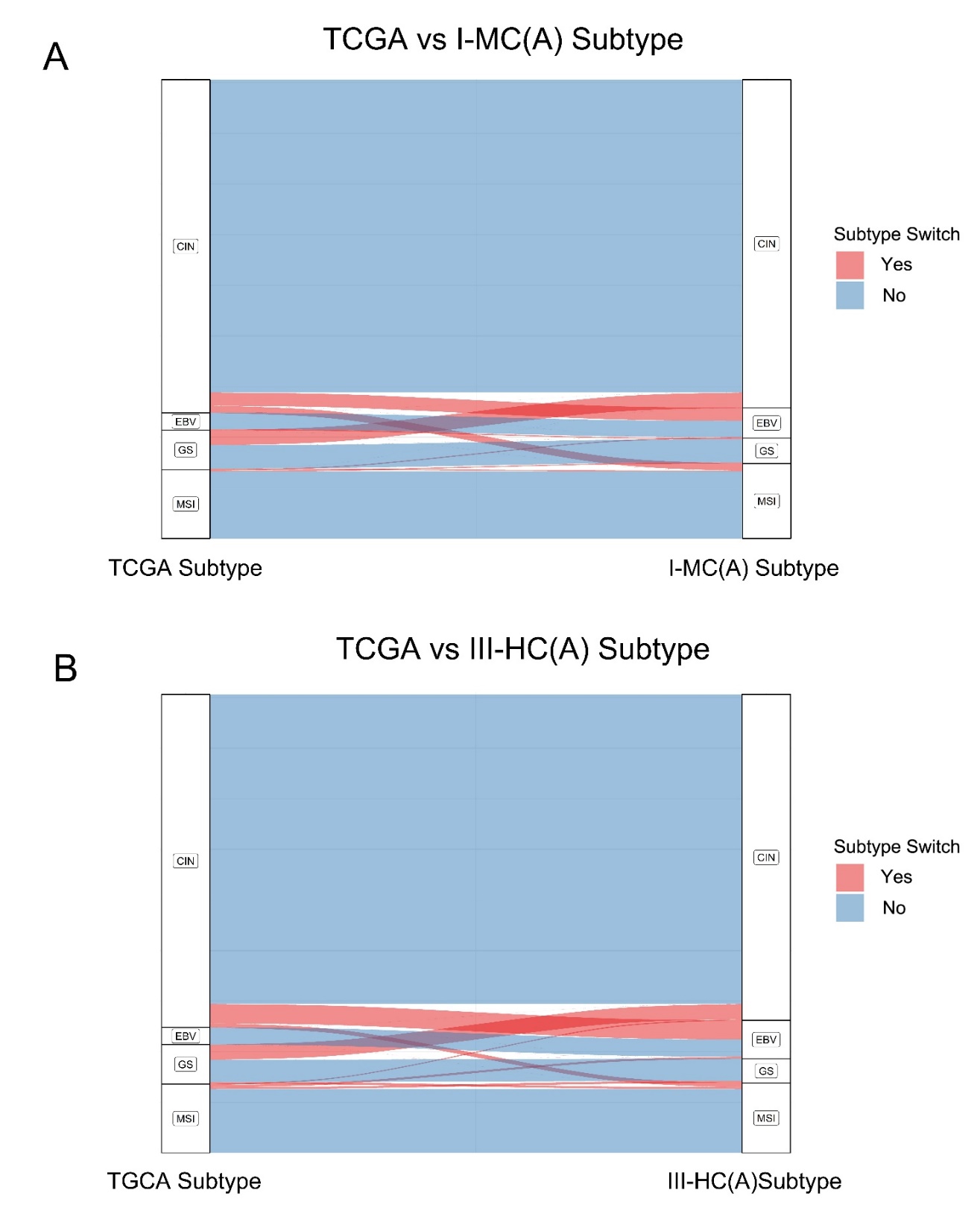
Supplementary Figure 1. Alluvial plots. (A)** Alluvial plot between TCGA subtypes and I-MC(A) subtypes. **(B)** Alluvial plot between TCGA subtypes and III-HC(A) subtypes.

**2. Supplementary Tables**

**Supplementary Table 1.** Accuracy, F1 score and auPRC for classifiers with single alteration.

| **Classifiers** | **Subtypes** | **Accuracy** | | **F1-score** | | **auPRC** | |
| --- | --- | --- | --- | --- | --- | --- | --- |
|  |  | **score** | **Overall score** | **score** | **Overall score** | **score** | **Overall score** |
| I-MC(G) | CIN | 0.85 | 0.82 | 0.90 | 0.71 | 0.98 | 0.84 |
|  | GS | 0.60 |  | 0.53 |  | 0.61 |  |
|  | MSI | 1.00 |  | 0.84 |  | 1.00 |  |
|  | EBV | 0.83 |  | 0.56 |  | 0.78 |  |
| I-MC(C) | CIN | 0.92 | 0.67 | 0.94 | 0.59 | 0.98 | 0.55 |
|  | GS | 0.20 |  | 0.26 |  | 0.28 |  |
|  | MSI | 0.74 |  | 0.62 |  | 0.57 |  |
|  | EBV | 0.83 |  | 0.53 |  | 0.35 |  |
| I-MC(M) | CIN | 0.87 | 0.82 | 0.91 | 0.78 | 0.96 | 0.81 |
|  | GS | 0.67 |  | 0.50 |  | 0.50 |  |
|  | MSI | 0.74 |  | 0.70 |  | 0.78 |  |
|  | EBV | 1.00 |  | 1.00 |  | 1.00 |  |
| II-HC(G) | CIN | 0.90 | 0.82 | 0.93 | 0.80 | 0.96 | 0.79 |
|  | GS | 0.60 |  | 0.47 |  | 0.62 |  |
|  | MSI | 0.95 |  | 0.97 |  | 0.79 |  |
|  | EBV | 0.83 |  | 0.83 |  | 0.79 |  |
| II-HC(C) | CIN | 0.77 | 0.56 | 0.86 | 0.40 | 0.96 | 0.42 |
|  | GS | 0.00 |  | 0.00 |  | 0.22 |  |
|  | MSI | 0.79 |  | 0.48 |  | 0.28 |  |
|  | EBV | 0.67 |  | 0.27 |  | 0.22 |  |
| II-HC(M) | CIN | 0.85 | 0.79 | 0.89 | 0.75 | 0.95 | 0.75 |
|  | GS | 0.67 |  | 0.45 |  | 0.42 |  |
|  | MSI | 0.63 |  | 0.67 |  | 0.61 |  |
|  | EBV | 1.00 |  | 1.00 |  | 1.00 |  |
| III-HC(G) | CIN | 0.90 | 0.75 | 0.93 | 0.76 | 0.97 | 0.71 |
|  | GS | 0.67 |  | 0.49 |  | 0.52 |  |
|  | MSI | 0.95 |  | 0.95 |  | 1.00 |  |
|  | EBV | 0.50 |  | 0.67 |  | 0.33 |  |
| III-HC(C) | CIN | 0.75 | 0.61 | 0.85 | 0.42 | 0.98 | 0.55 |
|  | GS | 0.00 |  | 0.00 |  | 0.13 |  |
|  | MSI | 0.84 |  | 0.47 |  | 0.54 |  |
|  | EBV | 0.83 |  | 0.34 |  | 0.54 |  |
| III-HC(M) | CIN | 0.86 | 0.79 | 0.89 | 0.76 | 0.96 | 0.79 |
|  | GS | 0.67 |  | 0.45 |  | 0.41 |  |
|  | MSI | 0.63 |  | 0.69 |  | 0.83 |  |
|  | EBV | 1.00 |  | 1.00 |  | 0.94 |  |

**Supplementary Table 2.** Multivariate survival analyses using all samples from TCGA and I-MC(A), II-HC(A) and III-HC(A) defined subtypes (Age and Sex).

| **Classifier** | **Factors** | **Characteristics** | **Count (%)** | **95% CI** | **Hazard Ratio** | **P-value^1^** |
| --- | --- | --- | --- | --- | --- | --- |
| TCGA | Age | <65 (Reference) | 188 (43.9%) | / | / | / |
|  |  | >=65 | 240 (56.1%) | (1.02, 1.93) | 1.39 | 0.036* |
|  | Sex | Female (Reference) | 126 (29.4%) | / | / | / |
|  |  | Male | 302 (70.6%) | (0.88, 1.80) | 1.25 | 0.218 |
| I-MC(A) | Age | <65 (Reference) | 188 (43.9%) | / | / | / |
|  |  | >=65 | 240 (56.1%) | (1.07, 2.00) | 1.45 | 0.017* |
|  | Sex | Female (Reference) | 126 (29.4%) | / | / | / |
|  |  | Male | 302 (70.6%) | (0.90, 1.80) | 1.27 | 0.171 |
| II-HC(A) | Age | <65 (Reference) | 188 (43.9%) | / | / | / |
|  |  | >=65 | 240 (56.1%) | (1.06, 2.0) | 1.44 | 0.021* |
|  | Sex | Female (Reference) | 126 (29.4%) | / | / | / |
|  |  | Male | 302 (70.6%) | (0.90, 1.80) | 1.28 | 0.168 |
| III-HC(A) | Age | <65 (Reference) | 188 (43.9%) | / | / | / |
|  |  | >=65 | 240 (56.1%) | (1.07, 2.04) | 1.46 | 0.017* |
|  | Sex | Female (Reference) | 126 (29.4%) | / | / | / |
|  |  | Male | 302 (70.6%) | (0.90, 1.80) | 1.28 | 0.168 |

^1^Statistical significance is based on the fitted multivariate cox model (log-rank test), * if P<0.05.
